# Medical Journal Revived

**Published:** 1873-08-15

**Authors:** 


Medical Journal Revived.—The *Peninsular Journal of Medicine*, which has been suspended for several years, is revived under the editorial management of Henry F. Lyster, M.D., of Detroit. It is a monthly, published at Detroit. The new issue commenced with the July number. We welcome it to our exchange list.

